# Divergent thinking in groups during cold-water immersion is impaired by cold stress not the cold shock response

**DOI:** 10.3389/fpsyg.2025.1512011

**Published:** 2025-02-12

**Authors:** Max Kailler Smith, Rebecca Weller, Tony Duong, Rebecca McClintock, Matthew Peterson, Nathaniel Barr, Douglas M. Jones, Timothy L. Dunn

**Affiliations:** ^1^Warfighter Performance Department, Naval Health Research Center, San Diego, CA, United States; ^2^Leidos Inc., San Diego, CA, United States; ^3^The School of Humanities and Creativity, Sheridan College, Oakville, ON, Canada

**Keywords:** divergent thinking, cold shock, cold stress, cold-water survival, anxiety, panic, pre-immersion preparedness

## Abstract

**Introduction:**

A primary hazard of working in cold maritime environments is the potential for a substantial man overboard situation in freezing waters. Sudden cold-water immersion (CWI) triggers the cold shock response (CSR), which consists of cardiorespiratory responses that increase the chance of drowning. If cold shock response severity can be mitigated, life-saving actions must be taken within the first 10 min, as after this time frame drowning occurs due to cold incapacitation. To date, research shows that executive functioning is generally impaired by intense, acute stress, which implies the ability to think through potential actions to maximize survival would also be impaired by the cold shock response.

**Methods:**

To examine whether the severity of cold shock response impairs higher-level thinking in a group, 29 active duty service members completed a group format Divergent Association Task (DAT; 4–5 per group) prior to and during a 13-min cold-water immersion (water temperature: 1.3°C, air temperature: −2.7°C).

**Results:**

Results showed no relationship between cold shock response magnitude, indexed by peak heart rate, and DAT performance. However, results indicated that those with lower skin temperatures performed worse on the DAT.

**Discussion:**

Results suggest that the ability to engage in divergent thinking is relatively preserved in the critical ~10-min window although skin cooling may bias attention toward the cold stress impacting task performance. Furthermore, subjective reports of the severity of the initial gasp tracked with peak heart rate demonstrating potential utility of subjective responses in the absence of respiratory measurements.

## 1 Introduction

Operating in cold maritime environments can be perilous. Maritime disasters result from vessel damage, inclement weather, rough seas, human error, and collisions (Marchenko, 2013). Such incidents can lead to ships sinking, where large groups face extreme cognitive and physiological stress of unplanned immersion in near-freezing temperatures, and must rapidly devise solutions collectively in open waters to survive. Whole-body immersion in water becomes extremely dangerous when water temperatures drop to 15°C (Brooks, 2003; O’Loan et al., 2023). Upon immersion in cold water, the cold shock response (CSR) occurs, which is characterized by a large inspiratory gasp., hyperventilation, tachycardia, hypertension, and increased cardiac output (Cooper et al., 1976; Stocks et al., 2004). Accompanying these physiological responses can be intense psychological reactions such as heightened anxiety and panic (Barwood et al., 2018). While the cold shock response commonly lasts only up to 3 min, intense pain, freezing sensations, numbness, and loss of dexterity follow (Barwood et al., 2024; Bierens et al., 2016; Chen et al., 2010; Wittmers and Savage, 1917). All these factors can severely impair one’s ability to perform critical survival thinking and actions, which are most effective within the first 10 min of immersion.

Extensive research has explored the impact of cold stress on individual cognitive performance. Palinkas (2001) provides a comprehensive overview indicating that while simple cognitive tasks and response inhibition are generally maintained under cold stress, more complex cognitive functions—particularly those requiring significant demands on working memory and cognitive flexibility—are compromised. These findings align with research that has generally shown intense, acute psychological stress, induced by physiological or psychological manipulations, impairs executive functioning. In a meta-analytic review, Shields et al. (2016) found that stressor severity was a significant moderator predicting the relationship between greater psychological stress and greater working memory and cognitive flexibility impairment. In this meta-analysis, cold-water immersion (CWI) manipulations were classified as the most severe stressor, further underscoring the significance of cold-water immersion as a potent stressor that can negatively impact cognitive performance. Additionally, attention control and adaptive problem-solving have shown impairment under cold stress conditions (Dunn et al., 2022; Ellis, 1982; Ellis et al., 1985; Enander, 1987; Giesbrecht et al., 1993). Cheung et al. (2007) found that even mild skin cooling (~ −1°C) can impair attention, suggesting that cognitive performance can be disrupted even at lower levels of cold stress. Similarly, Coleshaw et al. (1983) demonstrated that low core body temperature adversely affects memory registration and reasoning speed, highlighting the broader cognitive impairments induced by cold exposure.

Part of the debilitating effects of cold-water immersion can be attributed to the severity of the anxiety symptoms individuals experience prior to and during cold-water immersion. Previous research has shown that acute anxiety associated with unplanned immersions increases the magnitude of physiological responses (e.g., cold shock response), thus elevating the risk of poor decision-making, cold injury, and death (Barwood et al., 2017). Moreover, psychological factors, such as pre-immersion anxiety, have been shown to interact with biophysical factors to contribute to individual differences in cold shock response magnitude (Barwood et al., 2018). Specifically, higher pre-immersion anxiety amplifies both the respiratory (i.e., initial gasp and hyperventilation) and cardiovascular (i.e., cardiac output) components of the cold shock response. Pre-immersion anxiety has also been shown to effectively negate any cold shock response habituation effects. Given working memory and cognitive flexibility are susceptible to psychological stress-related impairment, it is plausible that the psychological stress (i.e., panic and anxiety) induced by cold shock response could also be a source of performance degradation in addition to the experienced physiological stress.

The experienced intensity of any one of the initial physiological or psychological responses to cold-water immersion would seem to challenge one’s ability to perform life-saving actions, let alone enable one to think clearly enough to generate and evaluate promising survival plans. Despite the overwhelming nature of these responses, they do subside, providing a brief window of opportunity before severe motor control impairments set in. It is therefore crucial to understand how the initial physiological responses to cold-water immersion affect cognitive abilities associated with creative thinking that could be core to survival in a group context. Divergent thinking involves generating multiple solutions to open-ended problems and is a critical process in problem solving, especially in novel contexts or when faced with unexpected challenges (Sternberg et al., 2004). Divergent thinking is likely vital for personnel who may encounter high-stakes scenarios such as large group man overboard events in freezing waters, where the initial phases of effective problem-solving and collaboration involves generating useful and creative courses of action to maximize survival.

The current study assessed how the severity of cold shock response influenced performance on a group format divergent association task (DAT). The group format DAT was adapted from the original individual DAT developed by Olson et al. (2021), which asks participants to name 10 words that are as different from each other as possible in all meanings and uses of the words. We hypothesized that if divergent thinking ability is compromised by the degree of physiological stress induced by cold shock response, then individuals experiencing more severe cold shock response would have more difficulty generating divergent responses than those with a milder cold shock response, as indexed by the cardiovascular component (i.e., peak heart rate). Similarly, we hypothesized that individual impairments would negatively affect the group such that groups with individuals who had more severe cold shock responses would generate less divergent responses on average than groups containing individuals with milder responses. A secondary aim of this study was to contribute to the literature regarding the impact of psychological preparedness on the cold shock response. As noted, anxiety and panic increase cold shock response magnitude. We gathered responses concerning pre-immersion anxiety and mental preparations to further explore the relationship between pre-immersion experiences and physiological responses.

## 2 Materials and methods

### 2.1 Participants

A convenience sample of 29 military service members (2 Female/27 Male; age: 26 ± 5 years; height: 176 ± 7 cm; weight: 85.9 ± 13.6 kg) enrolled in a cold-weather medicine course volunteered to participate in this study. Twenty-five participants were native English speakers, three were native German speakers proficient in English, and one was a native Burmese speaker proficient in English.^1^ Participants’ age, height, weight, and body fat percentage were collected at the time of study enrollment prior to any cold-water immersion (see Table 1 for descriptives of body composition variables). Body fat percentage was determined using bioelectrical impedance analysis as measured by the InBody720 (InBodyUSA, Cerritos, CA). The InBody720 is a research grade model with high reliability and validity compared to dual-energy X-ray absorptiometry (McLester et al., 2020; von Hurst et al., 2016). Participant experience and familiarity with cold-water immersions was not measured during enrollment in the study. However, participants had completed a unit on cold-water survivability as a part of their cold-weather medicine course just prior to participating in the study. All participants provided informed consent in accordance with the Declaration of Helsinki, and the study was approved by the Institutional Review Board at the Naval Health Research Center, San Diego, CA (Protocol # NHRC.2024.0002). After consenting all participants, determining the total sample size, and confirming with course training and medical staff a reasonable immersion group size, participants were randomly assigned to five groups of five and one group of four using an order randomizer.

**Table 1:** Median demographic and body composition information by cold-water immersion group.

Group	*N*	Native language (N)	Age (years)	Height (cm)	Weight (kg)	Body Mass Index (kg/m^2^)	Body fat (%)
1	4	4-English	26 ± 7	178 ± 4	87 ± 11.8	27.1 ± 3.9	27.6 ± 13.4
2	5	4-English + 1-German	24 ± 12	177 ± 3	87.4 ± 13.3	29.7 ± 1.3	18.6 ± 1.3
3	5	4-English + 1-German	30 ± 1	181 ± 0	99.3 ± 11.2	29.0 ± 2.8	18.2 ± 3.9
4	5	4-English + 1-German	26 ± 2	183 ± 11	89.5 ± 1.8	28.1 ± 2.5	20.2 ± 10.2
5	5	5-English	22 ± 0	170 ± 6	74.7 ± 5.4	27.1 ± 3.6	19.4 ± 13.5
6	5	4-English + 1-Burmese	22 ± 2	170 ± 2	72 ± 3.4	23.9 ± 4.4	21.6 ± 4.4
Total	29		24 ± 7	177 ± 11	87.4 ± 21.4	28 ± 4.3	19.6 ± 7.9

Median ± Interquartile range.

### 2.2 Group format divergent association task

**Figure 1:**
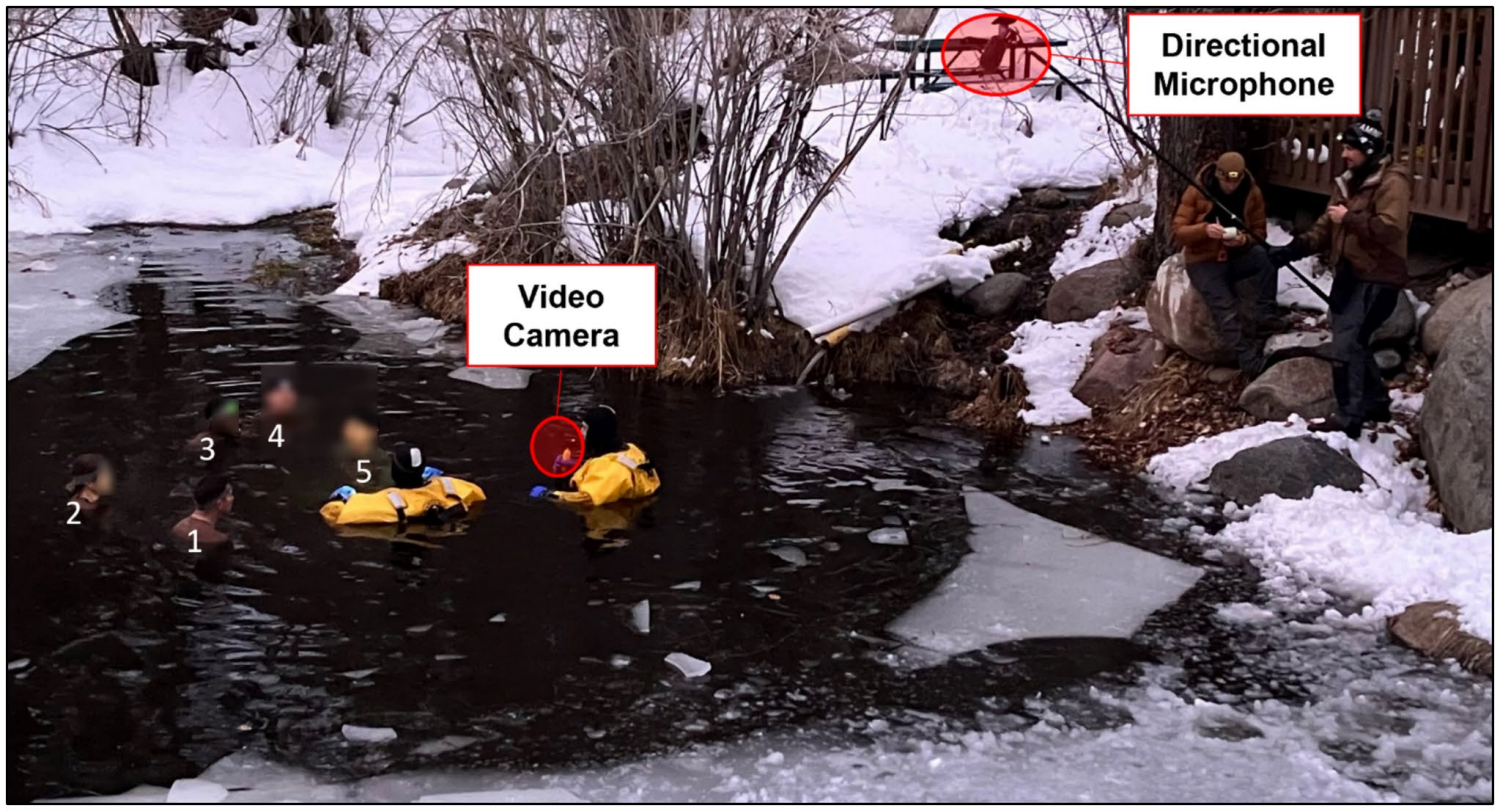
Picture of five participants (numbered 1–5 on the left side of the pond) performing the group format DAT. Participant responses were recorded with a video camera and directional microphone highlighted in red. Participants entered the water in the assigned task order. Participants generated responses in assigned order restarting with the first participant in the task order sequence after the last person in the sequence responded. Photo courtesy of Rebecca Weller at NHRC.

Participants completed the group format DAT twice, once during baseline testing in a thermoneutral indoor environment and once during outdoor cold-water immersion the following morning (see Figure 1). This task was chosen for its validation as a brief and reliable measure of divergent thinking, making it ideal for testing the impact of cold stress on creative cognition in a group. Ease of administration was also an important consideration given the constraints of the training exercise. The instructions, rules, and methods for the group format DAT closely followed the DAT developed by Olson et al. (2021) with some notable differences. For the group format DAT, participants were instructed to take turns one at a time in sequence saying out loud words that are as different as possible in all meanings and uses from the word stated prior by the previous group member. Participants were further told not to repeat words that they had individually already said or that were said by the group before them. Like Olson et al. (2021) participants were instructed to: (1) use only single words, (2) use only nouns, (3) avoid proper nouns, (4) avoid specialized vocabulary, and (5) think of words on your own and do not just name objects in your surroundings. If a participant responded with an invalid word (i.e., non-noun, proper noun, specialized vocabulary, or naming an object in surrounding environment), the group was instructed not to stop the task and continue generating valid words (see Supplementary material for Group Format DAT Instructions and Rules). The group format DAT persisted until all participants contributed 10 words (i.e., 50 total words for a group of 5).

### 2.3 Physiological measurements and subjective report

Heart rate was measured with a chest strap heart rate monitor (Polar Electro, Lake Success, NY). Peak heart rate ($HR_{\mathrm{peak}}$) within the first 6 min of the cold-water immersion was used to index the cardiovascular component of the cold shock response. To assess general cold stress, skin temperature sensors (Thermocron iButtons, iButtonLink Technology, Whitewater, WI) were placed on the chest, shoulder, thigh, hand (posterior), and foot (dorsal). Measurements from the chest, shoulder, and thigh were used to calculate mean skin temperature (${\bar{T}}_{sk}$) using the Burton equation (Ramanathan, 1964). Hand ($T_{\mathrm{hand}}$) and foot ($T_{\mathrm{foot}}$) skin temperatures were analyzed separately from (${\bar{T}}_{sk}$) given the relation between low extremity temperatures and worse attention control (Dunn et al., 2022). Participants ingested a core temperature ($T_{\mathrm{core}}$) capsule (BodyCap, Saint-Clair, France) for measuring gastrointestinal temperature. Temperature capsules were ingested approximately 8 h prior to the start of the cold-water immersion session. For all temperature measures, two variables were used in the subsequent analyses, absolute temperature at 6 min into immersion ($\mathrm{abs}T_{x})$ and change in temperature from min 0–6 of cold-water immersion ($\Delta T_{x}$). Finally, individual subjective experiences of the cold stress were gathered with a Post-Immersion Experience Questionnaire which consisted of 7 items (4 × 1–5 Likert Scale, 2 × Open-Ended response and 1 × Yes/No) asking participants to report on psychological and physiological experiences prior to and during the cold-water immersion (see Supplementary material for Post-Immersion Experience Questionnaire).

### 2.4 Experimental protocol

In assigned groups, participants completed the group format DAT in an auditorium (i.e., thermoneutral environment). Participants were given a temperature capsule and instructed to ingest the capsule at 2,100 that night. The next morning (~0600), participants were outfitted with a heart rate monitor and skin temperature sensors. The course instructors determined the length of the cold-water immersion based on the weather parameters of the morning. The cold-water immersion took place at an outdoor pond (elevation 2,100 m) and lasted 13 min in duration. Pond water temperature was 1.3°C, air temperature was −2.7°C, and wind speed was 0 m/s. The training requirements consisted of participants, in assigned groups, waiting to enter the pond (5 min) while outdoors, entering the pond (13 min; immersion to neck following brief head submersion), exiting the pond (5 min), changing into dry clothing, and completing rewarming until an instructor verified there was no longer a risk of cold injury.

The cold shock response subsides approximately 90 s to 3 min after initial immersion (Barwood et al., 2024; Bierens et al., 2016; Chen et al., 2010; Wittmers and Savage, 1917). Therefore, 3 min after entering the pond participants were instructed to begin the group format DAT. This allowed participants to experience the cold shock response and the associated physiological data to be collected before engaging in the group format DAT. A stream leading into the pond provided continuous slow current during all immersions. Additionally, instructors in the pond stirred the water by moving their arms back and forth around participants to ensure no beneficial boundary layer formed. Verbal responses were recorded with a directional microphone (Røde Videomic™) and a video camera (Go-Pro™). Participants wore standard-issue physical training gear consisting of a t-shirt, shorts, and shoes for entry into the water. After the cold-water immersion, participants changed into dry, cold-weather clothing (shirt, down jacket, down pants, down mittens, and down slippers) and could choose to enter a down sleeping bag or actively rewarm under the supervision of another member of the cold-weather medicine course. After successful rewarming (~1.5–2 h post immersion), participants completed the Post-Immersion Experience Questionnaire.

### 2.5 Analytic approach

Responses from the group format DAT were analyzed using Semantic Distance Analysis (SemDis). Semantic distance is the inverse of the cosine of the angle between vectors that correspond to each word within a given Euclidean semantic space (i.e., Semantic Distance = 1-Cosine Similarity; Landauer et al., 1998). A greater semantic distance value between two words therefore indicates the words are more unrelated. Semantic distance scores were computed using the GloVe algorithm (Global Vectors algorithm; https://nlp.stanford.edu/projects/glove) pretrained on the Common Crawl corpus (see https://hunspell.github.io for model corpus). Collected audio recordings of verbal responses were transcribed. All transcribed word responses were cleaned (i.e., compound words removed, spelling checked, etc.). Responses that did not appear in the chosen corpus were excluded from further analysis.

Four DAT scores were computed for the group format DAT, two group scores and two individual-within-group scores. To compute the group DAT score, the average of all the semantic distance scores calculated from all possible pairs of valid generated words by the group was taken, then multiplied by 100. The group DAT score aimed to capture a group’s overall performance on the DAT. That is, the ability to generate many words as different from each other in all meanings and uses. We also computed a group Sequence DAT score for each group. The group Sequence DAT score was computed in a similar manner to the DAT score, however the average of the semantic distance scores were only calculated between sequential word pairs rather than all possible pairs. For example, if three participants in sequence responded with the words “porridge”-“wheel”-“turban,” then the Sequence DAT score would be the average of the semantic distance scores for the “porridge”-“wheel” and the “wheel”-“turban” word pairs only. The group Sequence DAT therefore captures performance with consideration paid to the specific constraints imposed by producing verbal responses. That is, participants verbally responding imposed a sequential ordering to responding, whereas in the original version of the group DAT, participants could visually reference produced words as they typed words into 10 available slots. The group Sequence DAT represents the group’s ability to generate a word that is as different in all meanings and uses from the word that was generated by the group member before them.

The individual-within-group DAT score was computed by taking the average of all the semantic distance scores calculated from all possible pairs of valid generated words produced by a single individual within the group task. Similar to the group DAT score, this score reflects an individual’s ability to generate many semantically-distant words. The individual-within-group Sequence DAT score was computed by taking the average of the semantic distance scores between an individual’s responses and the words to which they were responding. For example, if participant A said “sock” then participant B said “aloe” in round 1, then in round 2 participant A said “story” and participant B said “cable,” the individual-within-group Sequence DAT score for participant B would be the average of the semantic distance scores for the “sock”-“aloe” and the “story”-“cable” word pairs. The individual-within-group Sequence DAT score better reflects an individual’s performance in consideration of the constraints imposed by responding in sequence to the prior word. That is, it represents an individual’s ability to generate a word response that is as different in all meanings and uses to what the person before them said.

Because we hypothesized that the magnitude of the cold shock response would negatively affect divergent thinking performance, we computed difference scores by subtracting baseline group and individual-within-group DAT scores from cold-water immersion group and individual-within-group DAT scores. Thus, the group DAT, group Sequence DAT, individual-within-group DAT, and individual within-group Sequence DAT used for analysis were all difference scores with negative values indicating worse performance during immersion and positive values indicating worse performance during baseline.

As a complimentary analysis to DAT scores, the frequency of task rule violations or repetitions of words during the DAT were counted at the individual- and group-level. We were particularly interested in rule violations that likely reflect impaired cognitive flexibility, executive control, or attentional biases since previous research has demonstrated that acute stress may impair these functions (Shields et al., 2016). For instance, naming objects in the environment or words that describe the stressor that is being experienced suggests a strong bias toward processing information directly related to the stressor rather than goal-directed spontaneous generation of dissimilar words. Likewise, repeating oneself, the word before them, or any words produced by others in the group similarly reflects cognitive fixedness. Finally, though participants were not explicitly told to refrain from repeating words they or others generated in the baseline session, considering repetitions of this kind provided important information regarding an inability to override strong, pre-potent concepts that were more readily to come to mind from the baseline session due to recent activation, use of sub-optimal strategies (i.e., “cheating/gaming” the task), or motivational state. For our analysis, sequence repetitions (i.e., repeating the word that was said by the person before you), self-repetitions (i.e., repeating a word that you already said), and naming objects in the immediate surrounding (e.g., rock, pond, tree, ice, etc.) were considered rule violations. Like the calculated DAT scores, a difference score of rule violations (i.e., subtracted baseline rule violations from cold-water immersion rule violations) was calculated to account for individual propensity to violate the stated rules.

Demographic data (age, height, weight, body mass index, body fat percent) were analyzed using Kruskal-Wallis tests to examine potential unintended differences between groups due to random assignment. Epsilon-squared (*ε*^2^) was calculated to estimate effect sizes for all Kruskal–Wallis tests. Significant group differences were followed up with *post hoc* pairwise Wilcoxon tests using a Bonferroni correction for multiple comparisons. We note here that group sizes were too small to conduct meaningful between-group statistics, though felt it was still important to present data related to potential unintended differences due to random assignment.

Linear regression analyses were used to test the relationships between cold shock response magnitude, indexed by $HR_{\mathrm{peak}}$, and DAT task variables (i.e., DAT, Sequence DAT, and task rule violations) at the individual-within-group and group levels. Due to lose contact of the sensor during immersion, we failed to collect heart rate data from three participants (i.e., one participant from Groups 3, 4, and 5). Therefore, all analyses that include $HR_{\mathrm{peak}}$ were performed excluding these participants. Kruskal-Wallis tests were used to examine group differences in $HR_{\mathrm{peak}}$ and group DAT task variables. Furthermore, a Wilcoxon signed rank test was conducted to test differences in total task time between the baseline and cold-water immersion sessions across all groups. Chi-squared analysis was used to further evaluate whether participants named objects in the environment or repeated previous responses during cold-water immersion at a significantly higher rate than expected given a reasonable expected rate indicating an effect of cold stress on DAT performance.

Spearman correlations were performed to assess the relationships between subjective ratings from the Post-Immersion Experience Questionnaire as well as to cold shock response magnitude, indexed by $HR_{\mathrm{peak}}$, and DAT performance. Exploratory analyses were also conducted to investigate the relationship between individual differences in skin and core temperatures and DAT performance. For these analyses, we performed four separate multiple regressions with either absolute skin temperatures ($\mathrm{abs}T_{x})$ or change in skin temperatures ($\Delta T_{x})$as predictors of individual-within-group DAT or Sequence DAT scores. If statistical diagnostics revealed multiple regression assumptions were violated, then appropriate tests or corrections were employed. Due to a logistical constraint of not having enough sensors, we failed to collect skin temperature data from five participants (three participants from Group 5 and two participants from Group 6). For these exploratory analyses, participants missing skin temperature data were excluded. We also performed four separate linear regression analyses to examine the relationships between core temperature variables ($\mathrm{abs}T_{c}\;\mathrm{or}\;\Delta T_{c})$ and individual-within-group DAT or Sequence DAT scores. Core temperature was not collected from four participants (i.e., one participant from Groups 1, 2, 3, and 5) because participants decided to not take the pill, or the pill was passed before data collection.

## 3 Results

### 3.1 Demographics

Kruskal–Wallis tests revealed groups differed in age [*χ*^2^(5) = 12.15, *p* = 0.032, *ε*^2^ = 0.31] and weight [*χ*^2^(5) = 11.22, *p* = 0.047, *ε*^2^ = 0.27]. However, follow-up pairwise Wilcoxon tests with Bonferroni correction did not identify any specific pairwise group differences for age (*p* > 0.05) as well as weight (*p* > 0.05). This suggests that differences in age and weight may be distributed across multiple groups rather than isolated to specific group comparisons. Height [*χ*^2^(5) = 9.53, *p* = 0.089, *ε*^2^ = 0.20] and body fat [*χ*^2^(5) = 2.74, *p* = 0.73, *ε*^2^ = 0.097] did not differ across groups. Critically for the purposes of this study, we confirmed sample groups did not differ in terms of key body composition variables (see Table 1 for descriptives of body composition variables).

### 3.2 Cold shock response magnitude and divergent thinking

**Figure 2:**
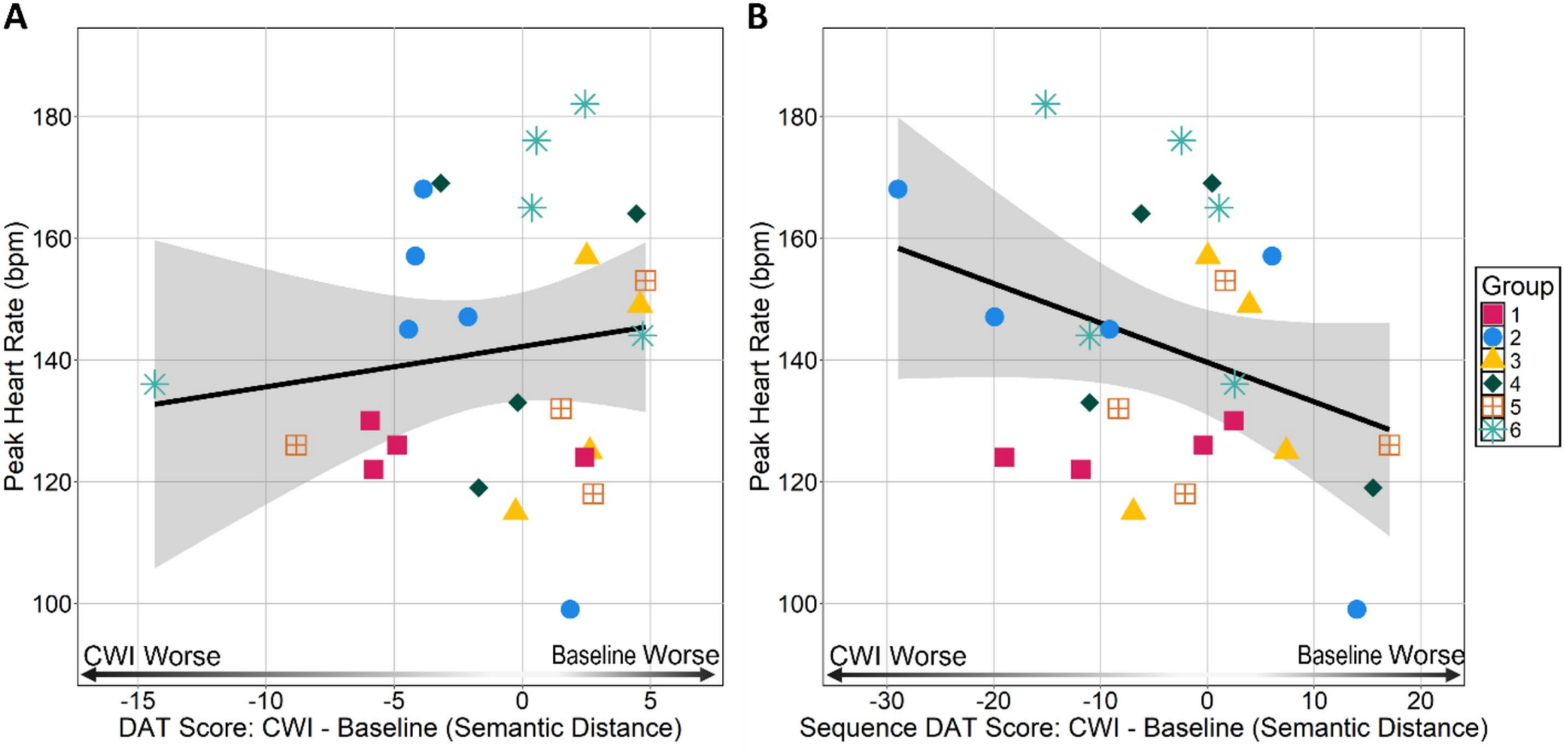
Peak heart rate $\left(HR_{\mathrm{peak}}\right)$ during immersion by **(A)** individual-within-group DAT and **(B)** individual-within-group Sequence DAT scores. DAT and Sequence DAT scores are difference scores calculated by subtracting an individual’s baseline score from their cold-water immersion (CWI) score. Negative values indicate an individual had a lower CWI score than baseline score and positive values indicate an individual had a lower baseline score than CWI score.

Our central research question concerned whether the magnitude of an individual’s cold shock response impacts divergent thinking ability. Individual divergent thinking ability was determined by two scores, individual-within-group DAT and individual-within-group Sequence DAT scores. We did not find a relationship between $HR_{\mathrm{peak}}$and individual-within-group DAT scores (*t*_24_ = 0.717, *p* = 0.48, *r* = 0.14, *r*^2^ = 0.021; see Figure 2A). We also did not observe a relationship between $HR_{\mathrm{peak}}$and individual-within-group Sequence DAT scores (*t*_24_ = −1.74, *p* = 0.09, *r* = −0.33, *r*^2^ = 0.11; see Figure 2B). Together these findings suggest that the severity of an individual’s cold shock response did not impact their ability to generate remote associations in general or in response to what was said before them.

**Figure 3:**
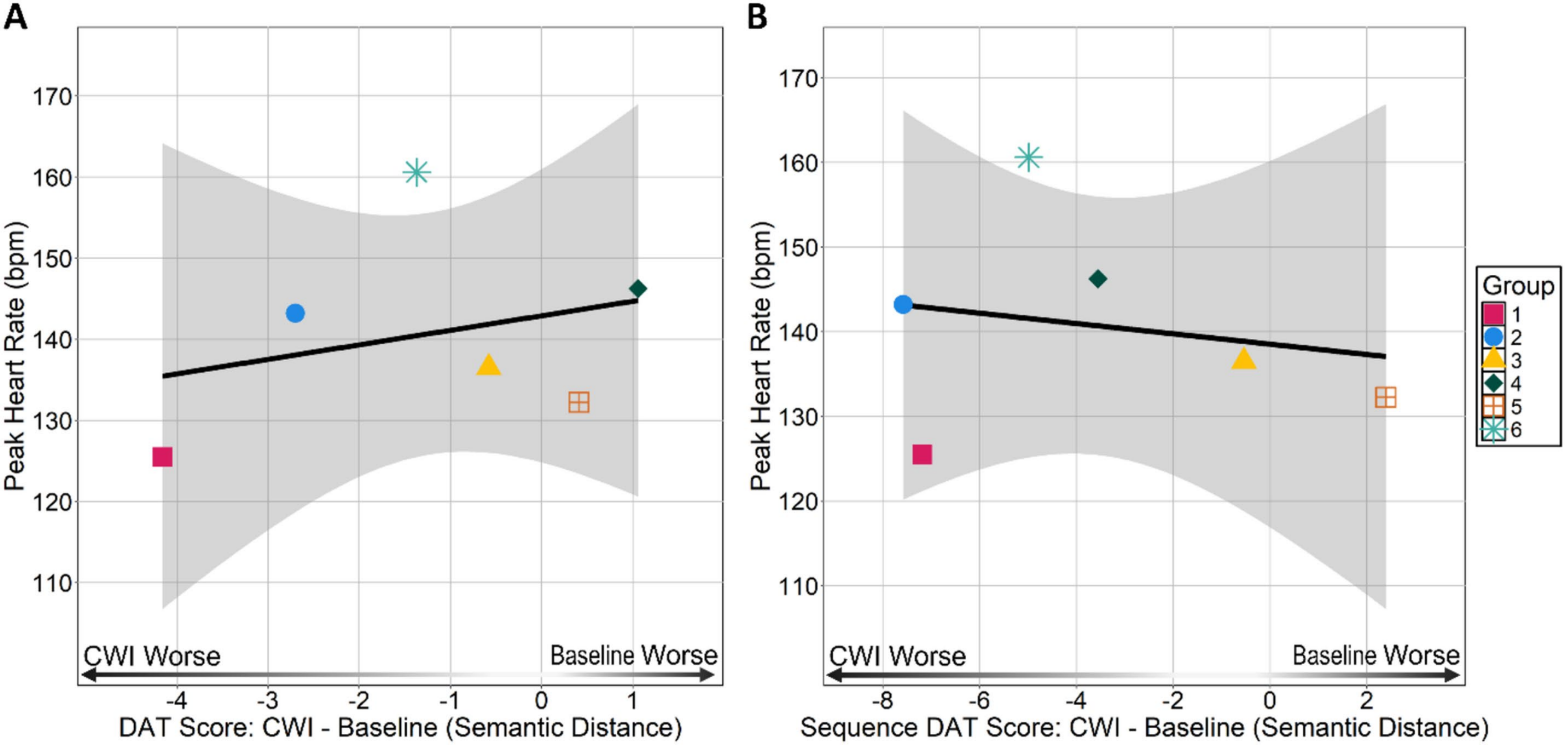
Peak heart rate $\left(HR_{\mathrm{peak}}\right)$ during immersion by **(A)** group average DAT and **(B)** group average Sequence DAT scores. DAT and Sequence DAT scores are difference scores calculated by subtracting a group’s baseline score from their cold-water immersion (CWI) score. Negative values indicate a group had a lower cold-water immersion score than baseline score and positive values indicate an individual had a lower baseline score than cold-water immersion score.

Since the DAT was performed in a group format, we were interested in identifying any between group differences in $HR_{\mathrm{peak}}$ and DAT performance (see Supplementary Table S1 for descriptives of $HR_{\mathrm{peak}}$ and DAT scores). A Kruskal–Wallis test did not reveal any differences between groups in $HR_{\mathrm{peak}}$ [*χ*^2^(5) = 7.65, *p* = 0.17, *ε*^2^ = 0.11] indicating groups did not differ in terms of their $HR_{\mathrm{peak}}$. We did not find any between group differences in individual-within-group DAT [*χ*^2^(5) = 5.38, *p* = 0.27, *ε*^2^ = 0.017] or individual-within-group Sequence DAT scores [*χ*^2^(5) = 3.17, *p* = 0.67, *ε*^2^ = 0.08]. These findings suggest that groups did not differ in terms of changes in their group member’s ability to generate remote associations in general or in response to what was said before them between baseline and cold-water immersion sessions. Additionally, at baseline individual-within-group DAT [*χ*^2^(5) = 7.44, *p* = 0.19, *ε*^2^ = 0.11] and individual-within-group Sequence scores [*χ*^2^(5) = 4.72, *p* = 0.45, *ε*^2^ = 0.012] did not differ ruling out potential group differences in ability to generate divergent responses in general in sequence at baseline. In line with the individual-within-group results, Spearman rank correlations revealed no relationship between group $HR_{\mathrm{peak}}$and group DAT scores (*ρ* = 0.31, *p* = 0.56; see Figure 3A) and no relationship between group $HR_{\mathrm{peak}}$and group Sequence DAT scores (*ρ* = 0.14, *p* = 0.80; see Figure 3B). Though these group-level correlations should be interpreted with caution given the small sample size, the results suggests there are no emergent group level relationships between cold shock response severity and divergent thinking performance.

Using a Wilcoxon singed rank test, we did find that groups tended to complete the DAT faster during cold-water immersion compared to baseline, *V* = 21, *p =* 0.035. At baseline, we found the group format DAT was completed in 95.5 s (Median = 94, IQR = 19), whereas during cold-water immersion groups completed the task in 69.16 s on average (Median = 65, IQR = 22).

**Figure 4:**
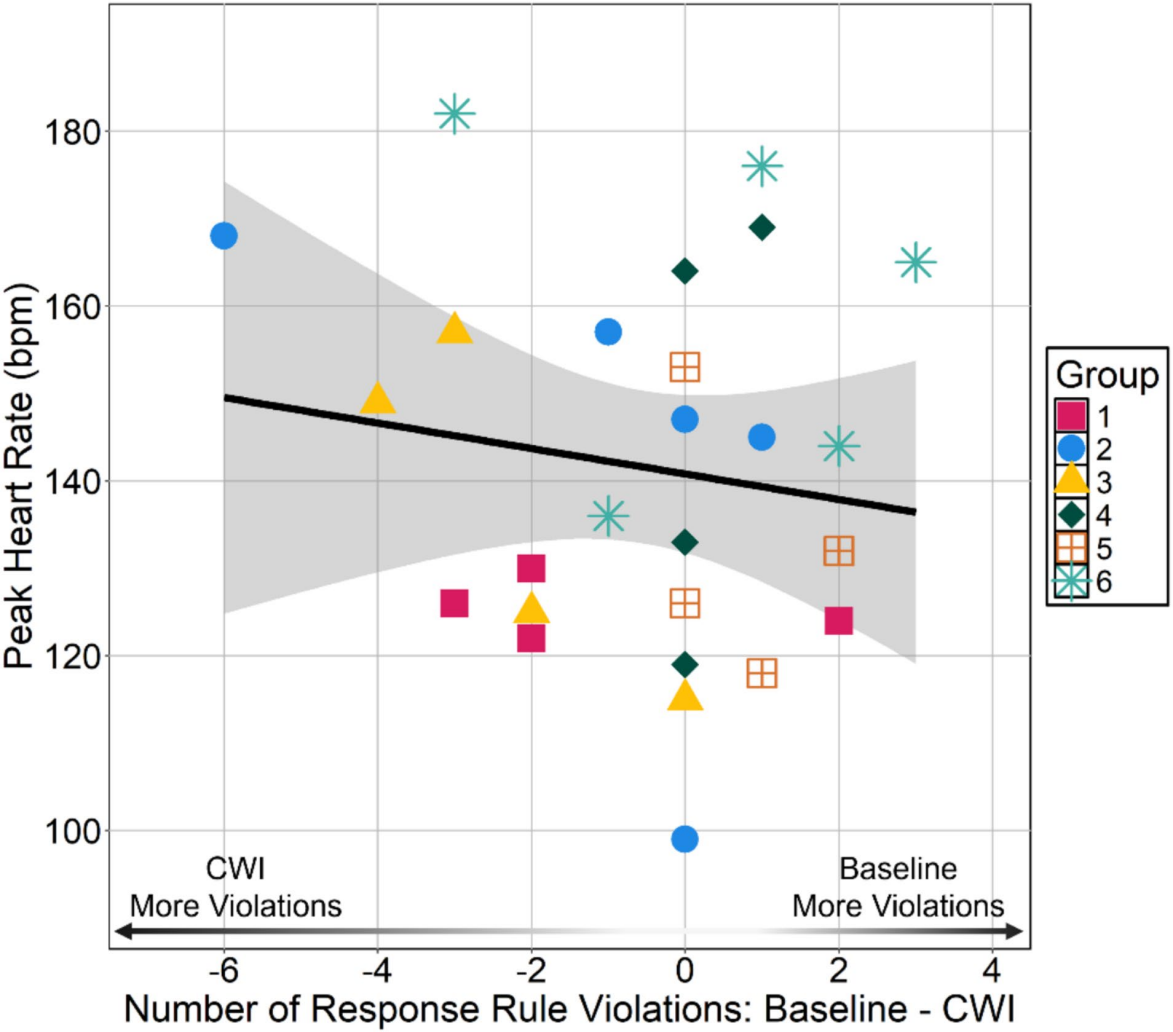
Peak heart rate $\left(HR_{\mathrm{peak}}\right)$ during immersion by difference in number of responses that violated task rules between cold-water immersion (CWI) and baseline DAT. Number of rule violation difference scores calculated by subtracting the number of responses that violated task rules at baseline DAT from the number of responses that violated task rules during cold-water immersion DAT. Negative values indicate an individual had more violations during the cold-water immersion DAT than the baseline DAT and positive values indicate an individual had more violations during the baseline DAT than the cold-water immersion DAT.

We analyzed the frequency of responses that violated task rules (see Supplemental material for Task Rules). We did not find a relationship between $HR_{\mathrm{peak}}$and individual-within-group frequency of responses that violated the task rules (*t*_24_ = −0.710, *p* = 0.48, *r* = −0.14, *r*^2^ = 0.020; see Figure 4). We also did not find a relationship between $HR_{\mathrm{peak}}$ and individual-within-group frequency of rule violations when considering cold-water immersion responses only (*t*_24_ = −0.10, *p* = 0.92, *r* = 0.02, *r*^2^ = 0.0004).

**Figure 5:**
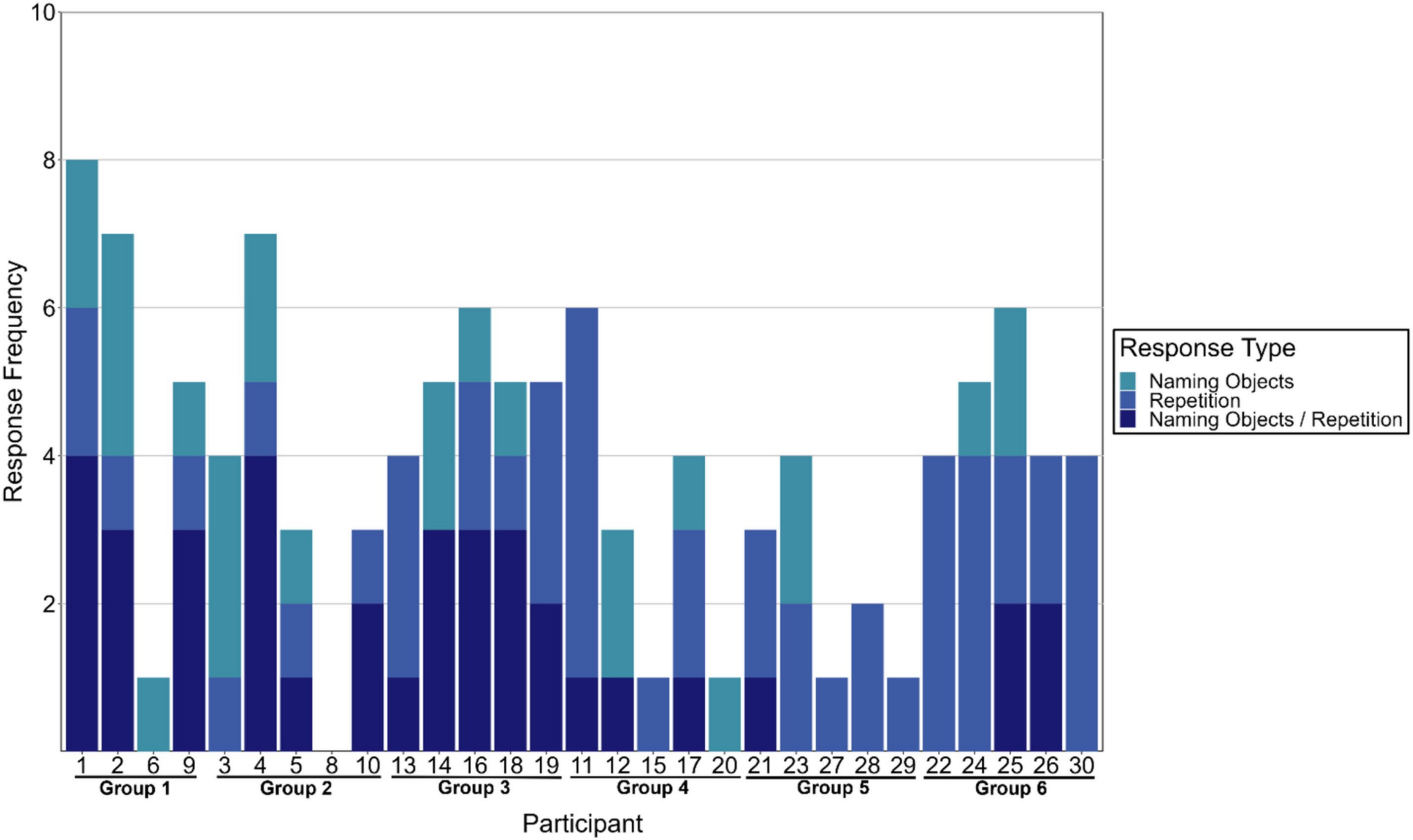
Number of responses out of 10 total generated during cold-water immersion DAT that were named objects in the surrounding and repetitions of what had already been said. The combination category “Naming Objects/Repetition” indicates ambiguity concerning whether the response was a named object, repetition, or both.

Repeating a word during the baseline DAT or repeating words generated by other group members during the cold-water immersion DAT was not considered a task rule violation since participants were not instructed to refrain from responding with these types of repetitions. Nonetheless, these types of technically valid repetitions may still indicate impaired cognitive flexibility or cognitive control since repeating what was already said reflects recall of an available word rather than goal-directed spontaneous generation of dissimilar words. Figure 5 depicts the number of responses out of the 10 given by each participant that were either named objects in the immediate environment or repetitions (i.e., sequence, self, self from baseline DAT, and group). We also identified responses could belong to either category, however since we did not ask participants to categorize their own responses, we were unable to resolve this ambiguity. After consideration of the additional forms of repetitions, we found that participants on average responded with either a named object in their surrounding or repeated what they or others said for 3.8 out of 10 total responses, though there was variability across participants (*SD* = 2.06 words). It is noteworthy, however, that 11 out of 29 participants responded with either a named object or a repetition for at least half of their responses. We performed a Chi-squared test to evaluate whether the rate of naming objects in the surroundings or repeating previous responses was higher than expected given specific instructions to avoid such behaviors. The expected rate for these types of responses was set at 10%, given participants were explicitly told not to name objects in their surroundings or repeat what they or the person before them had said. Despite these instructions, at baseline participants on average responded with a named object in their surrounding 1.8 out of 10 total responses (SD = 1.13), with only 2 out of the 29 participants (6.8% of participants) naming objects for 4 of the 10 total responses. Given this observed baseline rate for named objects and the explicit instructions, we determined 10% is reasonable as a conservative expected rate. The observed frequencies were compared to the expected frequencies under the null hypothesis. The contingency table is shown in Table 2.

**Table 2:** Contingency table – named objects/repetitions.

	Named objects/repetition (> = 5)	Named objects/repetition (<5)	Total
Observed	11	18	29
Expected	2.9	26.1	29

The chi-squared test revealed a statistically significant difference between the observed and the expected frequencies, *χ*^2^(1,29) = 25.13, *p* < 0.001. This indicates that the participants named objects in the environment or repeated previous responses at a significantly higher rate than the 10% baseline expectation.

A Kruskal–Wallis test was performed to evaluate any potential group differences that would indicate that some groups performed worse than others in this regard. We did not find any between group differences in number of responses that were named objects in the surrounding or a repetition of what was said before [*χ*^2^(5) = 9.47, *p* = 0.091, *ε*^2^ = 0.19].

### 3.3 Post-immersion experience questionnaire

Participants completed a Post-Immersion Experience Questionnaire after successful rewarming (~1.5–2 h post-cold-water immersion). With the responses gathered, we explored whether subjective ratings of the magnitude of the initial gasp during an individual’s cold shock response was correlated with $HR_{\mathrm{peak}}$. The questionnaire asked participants to rate the severity of their initial gasp upon entering the water using a 1–5 Likert scale with 1 indicating “No Gasp” and 5 indicating “Large Gasp.” We found higher subjective ratings indicating larger initial gasp reflex was correlated with higher $HR_{\mathrm{peak}}$, *ρ* = 0.43, *p* = 0.026. Participants also rated level of anxiety prior to entering the water, whether actual anxiety aligned with their expectations, and the degree of “panic” felt during immersion using 1–5 Likert scales. We did not find a relationship between level of anxiety prior to immersion and alignment with actual experienced anxiety during immersion *ρ* = −0.08, *p* = 0.66. However, higher ratings of anxiety did correlate with higher ratings of panic during immersion, *ρ* = 0.44, *p* = 0.017. Furthermore, those who reported higher degrees of panic during immersion also reported a larger gasp reflex, *ρ* = 0.59, *p* = 0.0009 and ratings of anxiety prior to immersion also were correlated with ratings of initial gasp reflex, *ρ* = 0.53, *p* = 0.002.

We also evaluated whether Post-Immersion Questionnaire responses correlated with DAT performance. Perceived magnitude of the initial gasp was not correlated with individual-within-group DAT scores (*ρ* = 0.001, *p* = 0.99) nor with Sequence DAT scores (*ρ* = −0.003, *p* = 0.98). Ratings of anxiety prior to entering the water also did not correlate with individual-within-group DAT scores (*ρ* = −0.1, *p* = 0.58) nor Sequence DAT scores (*ρ* = −0.1, *p* = 0.60). Furthermore, there were no relationships between ratings of perceived panic during immersion and individual-within-group DAT scores (*ρ* = −0.03, *p* = 0.85) or Sequence DAT scores (*ρ* = 0.11, *p* = 0.56).

### 3.4 Body temperatures and divergent thinking

Four regression models tested the relationship between skin temperatures ($\mathrm{abs}T_{x}\;\mathrm{or}\;\Delta T_{x})\text{,}$ as predictors of individual-within-group DAT scores (i.e., individual-within-group DAT or individual-within-group sequence DAT). The first linear regression model evaluated the relationship between $\mathrm{abs}T_{\mathrm{hand}}$, $\mathrm{abs}T_{\mathrm{foot}}$, and $\mathrm{abs}{\bar{T}}_{sk}$ as predictors of individual-within-group DAT scores (see Table 3). The second linear model evaluated the relationship between $\mathrm{abs}T_{\mathrm{hand}}$, $\mathrm{abs}T_{\mathrm{foot}}$, and $\mathrm{abs}{\bar{T}}_{sk}$ as predictors of individual-within-group Sequence DAT scores (see Supplementary Table S2). The third generalized additive model evaluated the relationship non-linear relationships between $\Delta T_{\mathrm{hand}}$, $\Delta T_{\mathrm{foot}}$, and $\Delta {\bar{T}}_{sk}$ as predictors of individual-within-group DAT scores (see Supplementary Table S3). A generalized additive model was used because diagnostic tests revealed violations of all multiple regression assumptions (i.e., linearity, normality of residuals, heteroscedasticity, and multicollinearity). Residual diagnostics of the GAM indicated assumptions of homoscedasticity and normality were reasonably met. The fourth linear regression model evaluated the relationship between $\Delta T_{\mathrm{hand}}$, $\Delta T_{\mathrm{foot}}$, and $\Delta {\bar{T}}_{sk}$ as predictors of individual-within-group Sequence DAT scores (see Supplementary Table S4).

**Table 3:** Model 1: linear regression of absolute skin temperature predictors on individual-within-group DAT scores.

Predictor	Estimate	95% CI		SE	*β*	*p*
		LL	UL			
Intercept	−10.99	−20.33	−1.66	4.47	0.17	0.023*
$\mathrm{abs}T_{\mathrm{hand}}$	−0.44	−1.02	0.93	0.46	−0.01	0.92
$\mathrm{abs}T_{\mathrm{foot}}$	0.18	−0.45	0.81	0.31	0.09	0.55
$\mathrm{abs}{\bar{T}}_{sk}$	0.93	0.08	1.80	0.41	0.37	0.034*

*F*(3,20) = 2.69, adjusted *r*^2^ = 0.18, *p* = 0.073. CI, confidence interval for Estimate; LL, lower limit; UL, upper limit; ${\mathrm{absT}}_{\mathrm{hand}}$, absolute hand skin temperature; ${\mathrm{absT}}_{\mathrm{foot}}$, absolute foot skin temperature; $\mathrm{abs}{\bar{T\;}}_{sk}$, absolute mean skin temperature. *p < 0.05.

**Figure 6:**
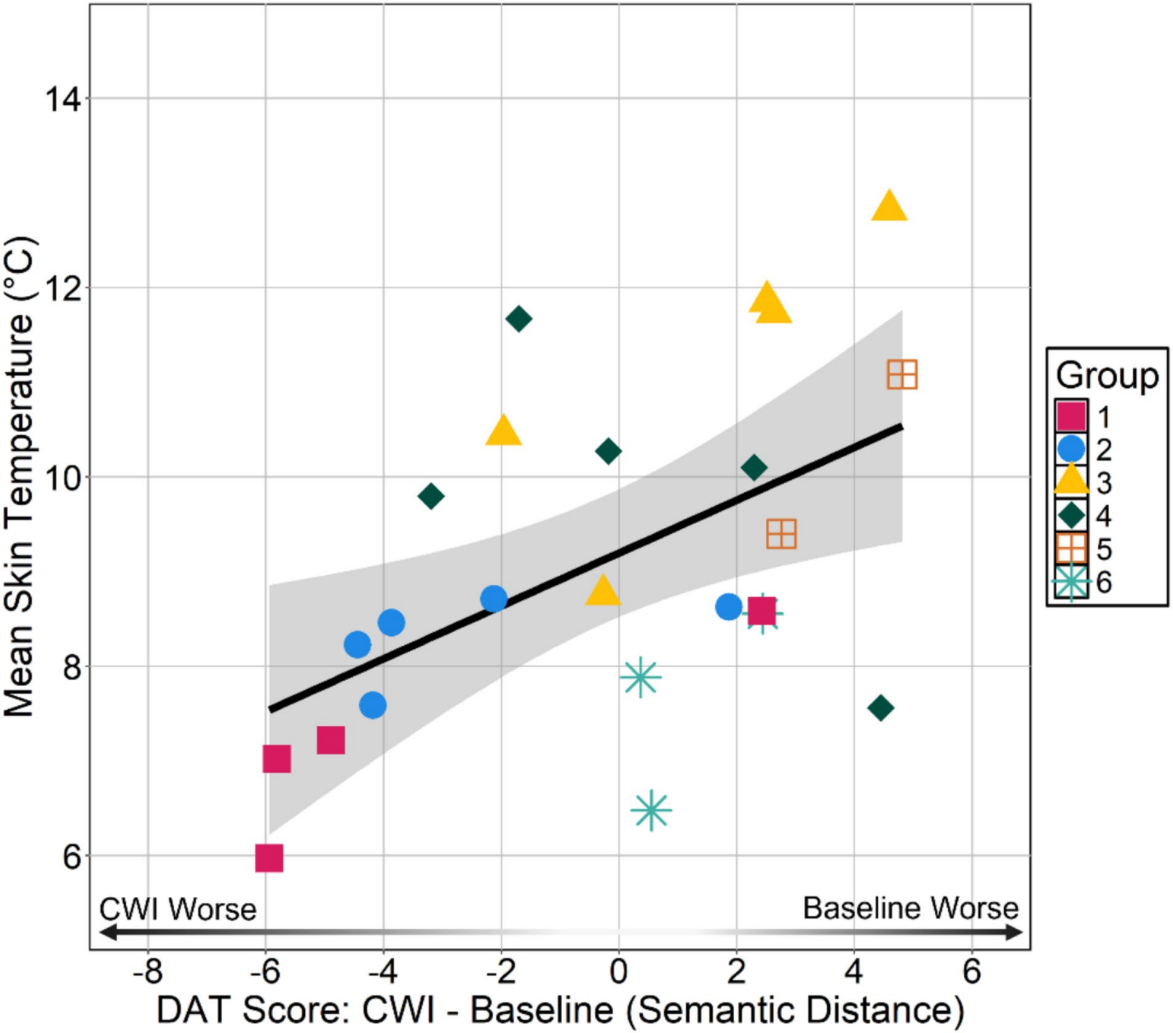
Absolute mean skin temperature $\left(\mathrm{abs}{\bar{T}}_{sk}\right)$ during immersion by individual-within-group DAT. DAT scores are difference scores calculated by subtracting an individual’s baseline score from their cold-water immersion (CWI) score. Negative values indicate an individual had a lower cold-water immersion score than baseline score and positive values indicate an individual had a lower baseline score than cold-water immersion score.

All models were not statistically significant indicating that the combination of skin temperature predictors did not explain a significant portion of the variance in individual-within-group DAT performance. However, post-hoc analysis of the individual predictors in the first linear regression model revealed that $\mathrm{abs}{\bar{T}}_{sk}$ was a significant positive predictor of individual-within-group DAT scores (see Table 3) suggesting that individuals with colder skin temperatures had lower DAT scores during immersion than at baseline (see Figure 6).

Individual differences in $T_{c}$ related to performance on the DAT was not expected as the duration of the cold-water immersion was too short to warrant substantial changes (Jones et al., 2023). Nonetheless, given these analyses were exploratory in nature, we wanted to identify whether there is evidence of any relationships between $T_{c}$ and DAT performance. We performed four separate linear regressions to test the relationship between core temperature and DAT performance. As predicted, we did not find any relationship between $T_{c}$ measures and DAT performance measures (see Supplementary Tables S5–S8).

## 4 Discussion

This study investigated the impact of the cold shock response on divergent thinking among group members in a field setting. We did not find evidence to suggest the magnitude of the cardiovascular component of cold shock response, indexed by peak heart rate ($HR_{\mathrm{peak}}$), subsequently impaired divergent thinking performance once the immediate effects of the cold shock response subsided. Specifically, there were no significant relationships between $HR_{\mathrm{peak}}$ and both individual DAT scores (i.e., individual-within-group DAT and Sequence DAT) nor with rule violations. Similarly, group-level analyses did not reveal any between groups differences in $HR_{\mathrm{peak}}$ or DAT scores suggesting there were no group-level effects that emerged despite observing no individual-level effects. Of note, groups completed the group format DAT faster during cold-water immersion compared to baseline, suggesting a potential effect of cold stress on task completion time. We also observed high rates of responses that were named objects in the environment and/or repetitions of what was already said (i.e., 11 participants >50% of cold-water immersion DAT responses). High rates of repeating words may account for why we observed no individual- or group-level differences in DAT performance between baseline and cold-water immersion sessions. Nonetheless, we did observe that absolute mean skin temperature $\left(\mathrm{abs}{\bar{T}}_{sk}\right)$ was correlated with individual-within-group DAT scores.

### 4.1 The impact of the cold shock response vs. cold stress on divergent thinking

Our key finding that the magnitude of the cardiovascular component of the cold shock response did not relate to divergent thinking performance in groups somewhat aligns with previous research suggesting that while cold stress can impair certain cognitive functions, it may not universally degrade all aspects of cognitive performance. For example, Palinkas (2001) noted that complex tasks requiring substantial cognitive flexibility and working memory are more likely to be compromised under cold stress, whereas simpler tasks may remain relatively unaffected. We had hypothesized divergent thinking would be impaired given its reliance on working memory and cognitive flexibility for enabling distant semantic associations to come to mind (Palmiero et al., 2022). However, in addition to executive processes, divergent thinking also relies on more spontaneous, associative processes (Barr, 2018; Beaty et al., 2014). It is possible generating remote associates was generally unaffected because participants leveraged a more associative style of responding at baseline and cold-water immersion. Moreover, response rate was faster during cold-water immersion which usually reflects greater response fluency or motivation (or lack thereof) (Wittmann and Paulus, 2008). However, as noted, there were also high rates of named objects/repetition responses compared to divergent word generation responses suggesting that participants defaulted quickly to responding with technically valid, yet suboptimal responses that allowed them to maintain decent DAT scores in the face of cold stress. Alternatively, it is possible that the task was performed during a relatively optimal time, post-cold shock response and prior to increased severity of other cold stress symptoms. We chose to administer the task during this critical time window because of its implications for providing useful guidance that considers what functions are preserved under cold-water immersion conditions. Buoite Stella and Morrison, 2022 demonstrated that even under milder cold water immersion conditions (18°C) performance on a task probing processing speed, selective attention, and working memory is degraded by the cold shock response. More generally, previous research showing cold stress cognitive impairment usually have participants experience cold stress for a longer period prior to task administration allowing cold stress symptoms to set in. Conversely, our results contribute to a more nuanced understanding by showing that once the cold shock response has subsided, divergent thinking—an essential component of creative problem solving—remains generally resilient. Together these findings suggests that upon initial immersion sailors should focus on surviving the cold shock response first, then orient to problem solving and planning, as opposed to trying to do both at the same time. Future research should investigate if there is a temporal threshold at which divergent thinking is impaired post-cold shock response to further qualify this guidance.

Despite finding no clear evidence that divergent thinking is significantly impaired by the cold shock response, the presented evidence is insufficient to claim that divergent thinking is completely unaffected. We observed the direction of the relationships of cold shock response magnitude to both individual-within-group Sequence DAT scores and rule violations were in line with the hypothesized direction, suggesting that with more power, or slightly more time, we could have observed a subtle effect. Over one-third of participants responded with objects in the immediate surroundings and/or repeated what was already said for at least half of their responses. One leading account of how stress impairs executive functioning that stress biases information processing to the immediate stressor (LeBlanc, 2009; Mather and Sutherland, 2011; Plessow et al., 2011). Given the nature of the DAT, participants could have responded with nearly any noun. Yet, a good portion of participants could not help but respond with words relating to the immediate stressor (e.g., “pond,” “ice,” “water,” “cold,” etc.). Similarly, it has been proposed that stress impairs cognitive control by shifting cognition from top-down control to automatic, bottom-up processing (Gagnon and Wagner, 2016; Vogel et al., 2016). Again, out of all possible words that would satisfy the task instructions, a good portion of participants chose to repeat what they or others had said suggesting fluency for repetitions given recent semantic activation. Together these findings indicate that while the cold shock response may not have negatively impacted divergent thinking performance in general, cold stress may bias attention toward processing the immediate stressor shifting cognition toward bottom-up processing. This aligns more generally with previous research showing that sympathetic activation (i.e., “fight or flight” response) constrains or narrows attention and thinking (Beilock and DeCaro, 2007; Easterbrook, 1959; Keinan, 1987; Shields et al., 2016). Furthermore, participants with lower mean skin temperatures had worse individual-within-group DAT scores. The saliency of cold skin temperatures was therefore likely the source of attention bias toward the cold stress comprising task performance. This finding aligns with Dunn et al. (2022) showing increased reaction times to a simple reaction time task during cold-water immersion suffer largely due to lower skin temperatures on the hand and the putative associated pain.

### 4.2 Subjective experience of cold-water immersion

Our analyses of subjective ratings to the Post-Immersion Experience Questionnaire provided additional insights into the role of psychological preparedness and its relationship with the cold shock response. The parameters of the field study did not allow us to obtain respiratory data to measure the magnitude of the respiratory component of everyone’s cold shock response (i.e., gasp reflex). However, we found subjective reports of the magnitude of the initial gasp reflex corresponded with $HR_{\mathrm{peak}}$. Although research specifically testing the relationship between subjective reports and objective measures of respiration are, to our knowledge, non-existent, this finding is encouraging as it suggests decent alignment between subjective ratings of initial gasp and the cardiovascular component of the cold shock response.

Despite the constraints the study environment posed to measuring the respiratory component of the cold shock response, the moderate correlation between subjective reports of initial gasp with $HR_{\mathrm{peak}}$ at least demonstrates that introspecting on the magnitude of one’s response aligns with measured responses of the cold shock response magnitude. Furthermore, although immersions were planned, we observed that participants’ psychological experiences prior to and during immersion influenced their physiological responses. Specifically, those who reported higher anxiety levels exhibited more pronounced cold shock response (i.e., higher cardiac and respiratory frequency and greater respiratory tidal volume) replicating previous work (e.g., Barwood et al., 2013, 2014, 2017, 2018). This relationship underscores the importance of psychological preparedness in managing physiological responses to stress. Barwood et al. (2013) highlighted that acute anxiety could amplify physiological stress responses, potentially impairing decision-making and increasing the risk of cold injury. Moreover, prior anxiety can effectively erase any positive adaptation effects gained from cold water habituation procedures (Barwood et al., 2017). These findings suggest that incorporating stress inoculation training, which includes controlled exposure to stressors and strategies for managing anxiety, could mitigate the adverse effects of acute stress on physiological and cognitive performance. Such training could enhance the overall resilience of personnel, enabling them to manage severe physiological responses to sudden immersion in near-freezing water.

### 4.3 Limitations and future directions

Given the constraints of performing research during an ongoing training exercise there were important limitations that should be considered. First, we did not measure the respiratory component of the cold shock response, which together with the cardiovascular measurements would have provided a more accurate measurement of the magnitude of the cold shock response. Second, while the group format DAT yielded rich response data in a very short period, including affording the opportunity to naturally observe instances of attentional bias and priming, we acknowledge that this task is not collaborative in nature nor indexes problem solving ability directly. Third, constraints of the training exercise prohibited us from administering the DAT during post-immersion rewarming period. Muller et al. (2012) have shown decline in working memory, choice reaction time, and executive functioning that persists 60 min into rewarming after 2 h of cold air (air temperature = 10°C) exposure. Alternatively, Jones et al. (2022) report partial recovery in psychomotor task performance at 60 min of passive-rewarming after a 10-min cold-water immersion (water temperature = 1°C). Future research should administer the DAT during rewarming, especially after skin temperatures recover to room temperature, to better understand whether attentional orienting to the stressor accounts for the divergent thinking impairment.

We also acknowledge that group sizes were too small to conduct meaningful between-group statistics. Even so, it was still important to report our group level findings to identify potential trends of interest for future research in this domain. Future work should focus on developing group problem solving tasks that are amenable to the logistical constraints of cold-water immersion. Additionally, the field setting of the study, while enhancing ecological validity, may have introduced uncontrolled variables affecting the outcomes. Though the training environment is much more realistic than cold-water immersion in a controlled laboratory environment, cold-water immersion was planned and therefore results may not fully germane to truly accidental cold-water immersion. Finally, we did not collect subjective report data prior to immersion. Retrospective ratings are susceptible to contamination due to known attention and memory based biases (e.g., priming, peak-end rule, hindsight, etc.). Future research investigating (mis)alignment between subjective experience prior to and during cold-water immersion and physiological responses to cold-water immersion should include more comprehensive questionnaires provided before and after cold-water immersion.

## 5 Conclusion

Our study contributes to the understanding of how the cold shock response affects cognitive performance, particularly divergent thinking. The findings suggest that while acute the magnitude of the cold shock response measured through the cardiovascular component does not strongly correlate with worse DAT performance, it does bias attention toward the immediate stressor. These insights can inform training and operational practices in cold environments, ensuring that certain cognitive functions are maintained, yet affected by cold stress. By highlighting the resilience of cognitive functions under stress and the importance of psychological factors, our study adds to the growing body of literature on how cold stress impacts cognitive performance.

Additionally, our exploratory findings on psychological preparedness and its influence on the cold shock response provide a foundation for developing targeted training programs that could enhance the resilience of seafarers and military personnel to cold stress. Future research should build on these findings to further understand the mechanisms underlying the relationship between acute stress, cognitive performance, and psychological preparedness, ultimately contributing to more effective strategies for managing cognition and physiology during extreme environmental stress.

## Data Availability

The datasets presented in this article are not readily available because of security protocols and privacy regulations, but they may be made available on reasonable request by the Naval Health Research Center Institutional Review Board. Requests to access the datasets should be directed to Phi Ngo, phi.h.ngo.civ@health.mil.

## Footnotes

^1^Proficiency in English by North American Treaty Organization (NATO) standards was a prerequisite for participation in the cold-weather medicine course.

## Author disclaimer

The views expressed in this article are those of the authors and do not necessarily reflect the official policy or position of the Department of the Navy, Department of Defense, nor the U.S. Government. I am a military service member or employee of the United States government. This work was prepared as part of my official duties. Title 17 U.S.C. 105 provides that copyright protection under this title is not available for any work of the United States Government. Title 17 U.S.C. 101 defines a U.S. Government work as work prepared by a military service member or employee of the U.S. Government as part of that person’s official duties.
