# Supplementary material for: Divergent thinking in groups during cold-water immersion is impaired by cold stress not the cold shock response
Source: Front Psychol. 2025 Feb 12;16:1512011. doi: 10.3389/fpsyg.2025.1512011 (PMC11860877; doi:10.3389/fpsyg.2025.1512011)
Supplement: Supplementary file 1 [file Data_Sheet_1.docx]

Supplementary Material

Group Format Divergent Association Task

**Instructions**

As a group you will take turns in sequence saying words that are as **different** as possible from the word said before you. That is, different in all meanings and uses. By the end of the task, each of you will have contributed 10 words total. When completing the task you must follow these rules.

**Rules**

1. You may not say your word until the person before you finished saying their word.
2. You may only say single words.
3. All words must be nouns (e.g., things, objects, concepts). No verbs, adverbs, adjectives, etc.
4. You may not say a proper noun (e.g., no specific people or places).
5. You may not use any specialized vocabulary (e.g., no technical terms).
6. You must think of words on your own (e.g., do not just look at objects in your surroundings).
7. Try not to repeat words that you have already said or was said by the person before you.

To begin the task, the experimenter will say the group number outload followed by the number of the participant who will begin. The experimenter will then say start. If your number is chosen, simply say the first noun that comes to mind. The person clockwise to the starter will then say their word based on the starter’s word and the task will continue in this manner until everyone has said 10 words.

Post-Immersion Experience Questionnaire

Please answer the following questions. You may skip questions you are not comfortable answering. There are additional questions on the reverse side of this questionnaire.

1. How anxious or nervous were you before entering the water (from the time you left the classroom and arrived at the staging area near the pond)?:


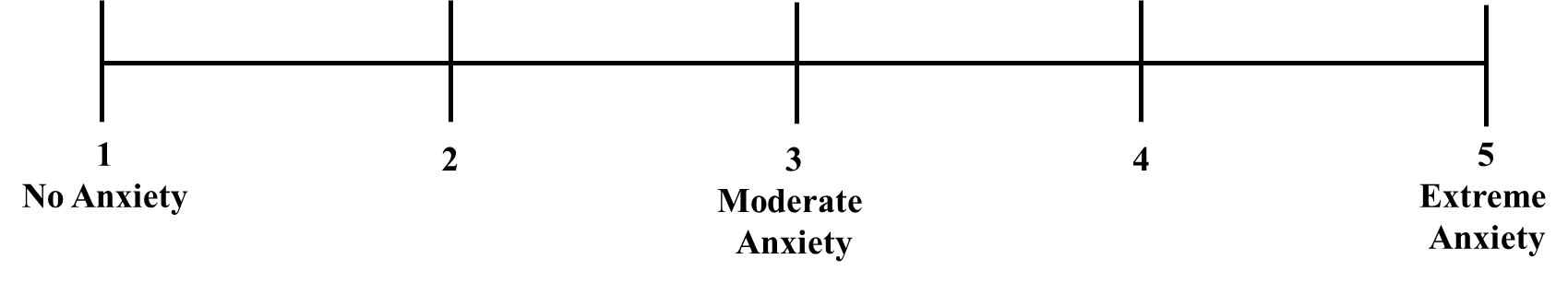


1. My actual anxiety during the cold-water immersion aligned with my expectations (from your rating above):


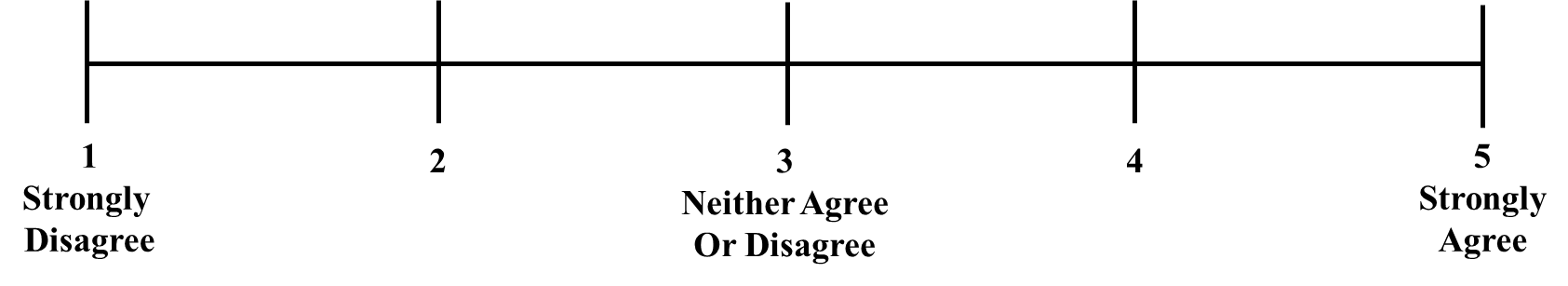


1. Was there anything you did to mentally prepare yourself before entering the water (open-ended)?
2. Did you experience an initial gasp in your breathing at the time you entered the water?


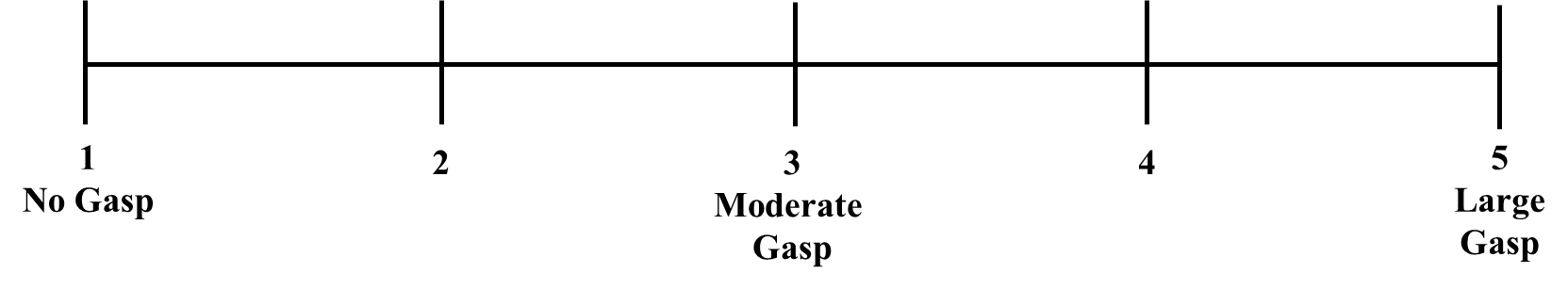


1. Were you able to control your breathing during immersion?
2. Yes
3. No
4. Please rate the degree of panic you felt during immersion:


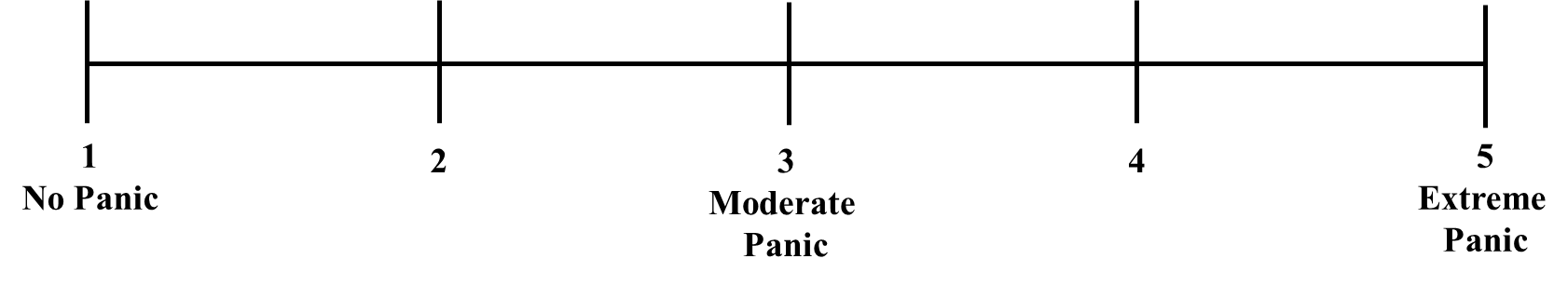


1. Was there anything specific you did while in the water to remain calm (open-ended)?

**Table 1-SM. Median Peak Heart Rate and Divergent Association Task Scores by Cold-Water Immersion Group**

| **Group** | **N** | $\boldsymbol{HR}_{\boldsymbol{peak}}$ **(bpm)** | | **Baseline DAT** | | **CWI DAT** | **DAT Difference Score** | **Baseline Sequence DAT** | **CWI Sequence DAT** | **Sequence DAT Difference Score** |
| --- | --- | --- | --- | --- | --- | --- | --- | --- | --- | --- |
| 1 | 4 | | 125 $\pm$ 4 | | 74.6 $\pm$ 2.3 | 70.1 $\pm$ 5.6 | -5.4 $\pm$ 2.8 | 75.8 $\pm$ 9.4 | 62.8 $\pm$ 11.7 | -6.1 $\pm$ 14.0 |
| 2 | 5 | | 147 $\pm$ 12 | | 80.3 $\pm$ 5.5 | 77.8 $\pm$ 5.9 | -3.7 $\pm$ 2.1 | 77.0 $\pm$ 8.7 | 67.9 $\pm$ 28.0 | -9.1 $\pm$ 26.0 |
| 3 | 5 | | 137 $\pm$ 29 | | 72.1 $\pm$ 4.9 | 74.8 $\pm$ 3.6 | 2.6 $\pm$ 1.3 | 72.7 $\pm$ 9.2 | 72.4 $\pm$ 5.2 | 0.1 $\pm$ 10.9 |
| 4 | 5 | | 148 $\pm$ 36 | | 76.0 $\pm$ 2.3 | 75.9 $\pm$ 5.6 | -1.0 $\pm$ 3.1 | 75.8 $\pm$ 2.5 | 69.6 $\pm$ 12.0 | -6.2 $\pm$ 11.5 |
| 5 | 5 | | 129 $\pm$ 13 | | 74.6 $\pm$ 5.9 | 76.4 $\pm$ 1.9 | 2.1 $\pm$ 4.4 | 68.5 $\pm$ 12.1 | 66.4 $\pm$ 8.1 | 1.7 $\pm$ 5.7 |
| 6 | 5 | | 165 $\pm$ 32 | | 75.5 $\pm$ 3.0 | 73.4 $\pm$ 6.1 | 0.6 $\pm$ 2.1 | 74.8 $\pm$ 2.0 | 71.9 $\pm$ 10.4 | -2.4 $\pm$ 12.1 |
| Total | 29 | | 140 $\pm$ 32 | | 75.5 $\pm$ 5.1 | 87.4 $\pm$ 5.3 | 0.1 $\pm$ 6.6 | 74.7 $\pm$ 9.1 | 69.6 $\pm$ 11.3 | -2.1 $\pm$ 13.6 |

Note: Median $\pm$Interquartile Range. ${HR}_{peak}$ = peak heart rate within first 6 minutes of the cold water immersion. All DAT scores reflect individual-within-group DAT scores. Difference scores = Baseline DAT – CWI DAT.

**Table 2-SM. Model 2: Linear Regression of Absolute Skin Temperature Predictors on Individual-Within-Group Sequence DAT Scores**

| **Predictor** | **Estimate** | **95% CI** | | **SE** | ***β*** | ***p*** |
| --- | --- | --- | --- | --- | --- | --- |
|  |  | **LL** | **UL** |  |  |  |
| Intercept | -14.73 | -48.10 | 18.63 | 15.99 | -0.08 | 0.37 |
| ${absT}_{hand}$ | 1.16 | -2.32 | 4.65 | 1.67 | 0.17 | 0.50 |
| ${absT}_{foot}$ | -0.53 | -2.81 | 1.73 | 1.09 | -0.11 | 0.62 |
| $abs\bar{T}_{sk}$ | 1.12 | -1.96 | 4.21 | 1.47 | 0.18 | 0.46 |

Note: *F(3,20)* = 0.64*,* adjusted *r*^2^ = -0.05, *p* = 0.60*.* CI = confidence interval for Estimate; LL = lower limit; UL = upper limit, ${absT}_{hand}$= absolute hand skin temperature, ${absT}_{foot}$= absolute foot skin temperature, $abs\bar{T}_{sk}$= absolute mean skin temperature. *p <.05

**Table 3-SM. Model 3: Generalized Additive Model of Change in Skin Temperature Predictors on Individual-Within-Group DAT Scores**

| **Predictor** | ***edf*** | **95% CI** | | **SE** | ***p*** |
| --- | --- | --- | --- | --- | --- |
|  |  | **LL** | **UL** |  |  |
| Intercept | -3.06 | -6.98 | 0.87 | 2.00 | 0.14 |
| ${\Delta T}_{hand}$ | 0.10 | -0.24 | 0.52 | 0.18 | 0.57 |
| ${\Delta T}_{foot}$ | 0.06 | -0.26 | 0.66 | 0.27 | 0.82 |
| $\Delta\bar{T}_{sk}$ | -0.38 | -0.23 | 0.91 | 0.35 | 0.28 |

Note: *N* = 24, *GCV* = 14.49*,* adjusted *r*^2^ = -0.027, *Deviance Explained* = 10.7%*.* edf = effective degrees of freedom, CI = confidence interval for *edf*; LL = lower limit; UL = upper limit, ${\Delta T}_{hand}$= change in hand skin temperature, ${\Delta T}_{foot}$= change in foot skin temperature, $\Delta\bar{T}_{sk}$= change in mean skin temperature. *p <.05

**Table 4-SM. Model 4: Linear Regression of Change in Skin Temperature Predictors on Individual-Within-Group Sequence DAT Scores**

| **Predictor** | **Estimate** | **95% CI** | | **SE** | ***β*** | ***p*** |
| --- | --- | --- | --- | --- | --- | --- |
|  |  | **LL** | **UL** |  |  |  |
| Intercept | -9.93 | -23.42 | 3.54 | 6.46 | -0.09 | 0.14 |
| ${\Delta T}_{hand}$ | -0.25 | -1.51 | 1.01 | 0.60 | -0.13 | 0.68 |
| ${\Delta T}_{foot}$ | -0.09 | -1.94 | 1.75 | 0.88 | -0.04 | 0.92 |
| $\Delta\bar{T}_{sk}$ | -0.23 | -2.58 | 2.11 | 1.12 | -0.10 | 0.83 |

Note: *F(3,20)* = 0.48*,* adjusted *r*^2^ = -0.07, *p* = 0.70*.* CI = confidence interval for Estimate; LL = lower limit; UL = upper limit, ${\Delta T}_{hand}$= change in hand skin temperature, ${\Delta T}_{foot}$= change in foot skin temperature, $\Delta\bar{T}_{sk}$= change in mean skin temperature. *p <.05

**Table 5-SM. Linear Regression of Absolute Core Temperature on Individual-Within-Group DAT Scores**

| **Predictor** | **Estimate** | **95% CI** | | **SE** | ***β*** | ***p*** |
| --- | --- | --- | --- | --- | --- | --- |
|  |  | **LL** | **UL** |  |  |  |
| Intercept | 80.48 | -30.63 | 191.60 | 53.72 | 0.12 | 0.15 |
| ${absT}_{core}$ | -2.18 | -5.17 | 0.81 | 1.44 | -0.30 | 0.14 |

Note: *t(23)* = -1.51*, r* = 0.29, *r*^2^ = 0.089, *p* = 0.14*.* CI = confidence interval for Estimate; LL = lower limit; UL = upper limit, ${absT}_{core}$= absolute core temperature. *p <.05

**Table 6-SM. Linear Regression of Change in Core Temperature on Individual-Within-Group DAT Scores**

| **Predictor** | **Estimate** | **95% CI** | | **SE** | ***β*** | ***p*** |
| --- | --- | --- | --- | --- | --- | --- |
|  |  | **LL** | **UL** |  |  |  |
| Intercept | -1.60 | -3.93 | 0.73 | 1.13 | 0.13 | 0.17 |
| ${\Delta T}_{core}$ | -3.59 | -8.03 | 0.85 | -1.67 | -0.33 | 0.11 |

Note: *t(24)* = -1.67*, r* = 0.32, *r*^2^ = 0.10, *p* = 0.10*.* CI = confidence interval for Estimate; LL = lower limit; UL = upper limit, ${\Delta T}_{core}$= change in core temperature. *p <.05

**Table 7-SM. Linear Regression of Absolute Core Temperature on Individual-Within-Group Sequence DAT Scores**

| **Predictor** | **Estimate** | **95% CI** | | **SE** | ***β*** | ***p*** |
| --- | --- | --- | --- | --- | --- | --- |
|  |  | **LL** | **UL** |  |  |  |
| Intercept | 122.67 | -158.46 | 403.86 | 135.91 | 0.04 | 0.38 |
| ${absT}_{core}$ | -3.37 | -10.95 | 4.18 | 3.66 | -0.20 | 0.37 |

Note: *t(24)* = -0.92*, r* = 0.18, *r*^2^ = 0.035, *p* = 0.36*.* CI = confidence interval for Estimate; LL = lower limit; UL = upper limit, ${absT}_{core}$= absolute core temperature. *p <.05

**Table 8-SM. Linear Regression of Change in Core Temperature on Individual-Within-Group Sequence DAT Scores**

| **Predictor** | **Estimate** | **95% CI** | | **SE** | ***β*** | ***p*** |
| --- | --- | --- | --- | --- | --- | --- |
|  |  | **LL** | **UL** |  |  |  |
| Intercept | -3.51 | -9.56 | 2.53 | 2.92 | 0.03 | 0.24 |
| ${\Delta T}_{core}$ | -1.54 | -13.09 | 9.99 | 5.58 | -0.06 | 0.78 |

Note: *t(24)* = -0.27*, r* = 0.057, *r*^2^ = 0.003, *p* = 0.78*.* CI = confidence interval for Estimate; LL = lower limit; UL = upper limit, ${\Delta T}_{core}$= change in core temperature. *p <.05
